# Dementia caregiving across Latin America and the Caribbean and brain health diplomacy

**DOI:** 10.1016/s2666-7568(21)00031-3

**Published:** 2021-03-31

**Authors:** Agustin Ibáñez, Stefanie Danielle Pina-Escudero, Katherine L Possin, Yakeel T Quiroz, Fernando Aguzzoli Peres, Andrea Slachevsky, Ana Luisa Sosa, Sonia M D Brucki, Bruce L Miller, on behalf of the Multi-Partner Consortium to Expand Dementia Research in Latin America

**Affiliations:** **Global Brain Health Institute, University of California San Francisco, San Francisco, CA, USA** (Prof A Ibáñez PhD, S D Pina-Escudero MD, K L Possin PhD, F A Peres MS, Prof B L Miller MD); **Global Brain Health Institute, Trinity College Dublin, University of Dublin, Dublin, Ireland** (Prof A Ibáñez, S D Pina-Escudero, K L Possin, F A Peres, Prof B L Miller); **Latin American Brain Health Institute, Universidad Adolfo Ibáñez, Santiago, Chile** (Prof A Ibáñez); **Cognitive Neuroscience Center, Universidad San Andres, Buenos Aires, Argentina** (Prof A Ibáñez); **CONICET, Buenos Aires, Argentina** (Prof A Ibáñez); **Department of Neurology, Memory and Aging Center, University of California San Francisco, San Francisco, CA, USA** (S D Pina-Escudero, K L Possin, Prof B L Miller); **Harvard Medical School, Massachusetts General Hospital, Boston, MA, USA** (Y T Quiroz PhD); **Geroscience Center for Brain Health and Metabolism, Memory and Neuropsychology Clinic, Neuropsychology and Clinical Neuroscience Laboratory, Physiopathology Department, and Neuroscience and East Neuroscience Department, Faculty of Medicine, University of Chile, Santiago, Chile** (Prof A Slachevsky PhD); **Neurology Service, Department of Medicine, Facultad de Medicina Clínica Alemana, Universidad del Desarrollo, Santiago, Chile** (Prof A Slachevsky); **National Institute of Neurology and Neurosurgery Manuel Velasco Suaréz, Mexico City, Mexico** (Prof A L Sosa PhD); **Cognitive and Behavioral Neurology Unit, University of São Paulo, São Paulo, Brazil** (Prof S M D Brucki PhD)

## Abstract

The prevalence of dementia in Latin America and the Caribbean is growing rapidly, increasing the burden placed on caregivers. Exacerbated by fragile health-care systems, unstable economies, and extensive inequalities, caregiver burden in this region is among the highest in the world. We reviewed the major challenges to caregiving in Latin America and the Caribbean, and we propose regional and coordinated actions to drive future change. Current challenges include the scarcity of formal long-term care, socioeconomic and social determinants of health disparities, gender-biased burdens, growing dementia prevalence, and the effect of the current COVID-19 pandemic on families affected by dementia. Firstly, we propose local and regional short-term strategic recommendations, including systematic identification of specific caregiver needs, testing of evidence-based local interventions, contextual adaptation of strategies to different settings and cultures, countering gender bias, strengthening community support, provision of basic technology, and better use of available information and communications technology. Additionally, we propose brain health diplomacy (ie, global actions aimed to overcome the systemic challenges to brain health by bridging disciplines and sectors) and convergence science as frameworks for long-term coordinated responses, integrating tools, knowledge, and strategies to expand access to digital technology and develop collaborative models of care. Addressing the vast inequalities in dementia caregiving across Latin America and the Caribbean requires innovative, evidence-based solutions coordinated with the strengthening of public policies.

## Introduction

The ageing population (older than 60 years) of Latin American and Caribbean countries (LACs) is growing rapidly,^1,2^ a demographic shift that will increase the already high social and economic burdens of dementia and caregiving in the region. Fragile health-care systems, unstable economic development, deficiencies in formal care, and large economic disparities are overburdening caregivers in LACs.^1–5^ Moreover, patients with dementia and their caregivers in LACs are being disproportionately affected by the COVID-19 pandemic,^6,7^ calling for additional coordinated efforts to mitigate the increased burden on these families. We review the key social and economic challenges of dementia caregiving in LACs and we discuss the possible trajectory of these challenges if not urgently addressed. Next, we present a set of short-term (3–5 years) goals to accelerate regional changes that might improve the experiences of caregivers. Finally, we propose long-term (3–10 years) strategies that could mitigate the projected burdens, by incorporating approaches, already shown in high-income countries, to be effective in mitigating the social and economic burdens of caregiving. We also summarise the key challenges to implementing these initiatives in LACs, and we call for an urgent coordinated collaborative plan that incorporates brain health diplomacy (figure).

## Current challenges of dementia caregiving in LACs

Caregiver burden is the perceived stress that results from the physical tasks, emotional demands, and restricted ability to socialise as a consequence of caring for a chronically ill person. Caregiver burden across LACs is among the highest in the world,^1,2,8^ and caregivers in LACs exhibit poorer mental health and quality of life than those in other regions.^8–13^ However, more systematic cross-regional comparisons of caregiver burdens are still urgently needed. The caregiver burden in LACs is exacerbated by the scarcity of formal long-term care systems, the enormous financial costs of caregiving, the negative socioeconomic factors associated with caregiving, and the strain and stigma associated with dementia and with caregiving The available evidence discussed in this section is summarised in the table.

Formal long-term care systems and regional policies for patients with dementia are almost non-existent in LACs.^14,31^ Long-term care is scantily covered by health insurance providers and is an underfinanced commodity in the region. Comprehensive public services are rare: only 1% of the population over the age of 60 years receives governmental support for long-term care,^14^ and only the wealthiest individuals and families in LACs can afford private long-term care. The economic impact of dementia in LACs is substantial. Including the expenses incurred by caregivers, the costs for each patient with dementia throughout the course of the disease (8 years on average) can be extremely high and exceed what most people living in LACs can afford, presenting an insurmountable financial burden for most people in the region. There are few home-care agencies, and daycare, domiciliary, or other long-term care facilities for advanced dementia care are accessible only to patients and families with sufficient financial resources to cover the high costs.^22,32^ Compensating for poor formal governmental support and insufficient financial resources in LACs,^29^ female caregivers and caregivers with low education typically spend 8–11 h per day providing informal care. This responsibility represents a high, indirect dementia-related cost for families.^18,21,26^ Formal caregivers are underpaid in most countries and only infrequently receive basic dementia training.^9,13^

The relationship between family socioeconomic status and caregiver strain in LACs is complex. Although a high caregiver burden is common across all socioeconomic strata in LACs,^18,21,26,29–31^ caregivers who report fewer household assets or who need to cut back on paid work to become a caregiver report a higher strain.^8^ Families in LACs shoulder a greater proportion of the costs of care than families in Europe and the USA,^19,21,26,29,30^ and lose more wages due to time spent caregiving. This economic strain results in multigenerational poverty as families’ financial resources are depleted during caregiving.^15^ Furthermore, caregivers have less opportunity to advance their own careers or to support the education or career advancements of their children. 56% of people with dementia in LACs have high rates of modifiable risk factors for dementia,^33^ such as low education and hypertension, compared with 35% globally.^34,35^ These same risk factors are present in dementia caregivers, increasing their vulnerability to physical comorbidities. The little assistance given to families by governments after the death of their family member with dementia is another relevant issue across LACs. Finally, the inadequacy of governmental support for families with patients living with dementia often generates financial difficulties that continue after the death of the patient, who might have been the only person in the home to have received a pension.

A cultural expectation in LACs is that family members will take care of relatives who are chronically ill, and dementia care in LACs is typically delivered informally within multigenerational households. Most patients with dementia remain in their homes, where their caregiver is a family member, friend, or neighbour who does not receive monetary compensation for the caregiving work.^36^ Female family members bear most of the caregiving burden,^12–15,22,27^ and are at a higher risk of depression and poorer physical health outcomes^8,37,38^ than male caregivers. Even patients with advanced dementia and substantial care needs are normally cared for at home until their death, contributing to high caregiver burden.^8,9,11,16,17,19,23,27,29^ In contrast to LACs, this burden is often mitigated in Europe and in the USA and Canada, where patients with high care needs are more likely to be transitioned to residential facilities or to receive other kinds of formal care.

Stigma and underdiagnosis of dementia create additional caregiving challenges. The substantial stigma associated with dementia across LACs^1,2,39,40^ (panel) creates barriers to diagnosis and care, infringes on the human rights of people with dementia, and exacerbates the burden for caregivers and patients.^8,41^ Dementia is not widely understood to be a disease in the region. Rather, ageing is conceived as a negative process associated with physical and mental decline.^40^ In LACs, dementia is often diagnosed late or is never diagnosed,^42^ which results in missed opportunities for care and planning. Dementia syndromes with prominent psychiatric and behavioural symptoms, including dementia with Lewy bodies^43^ and frontotemporal dementia,^44^ cause the most severe familial disruption and financial impact, but remain underdiagnosed and poorly studied in LACs,^19,24,30^ leaving families with little guidance to navigate this challenging territory.

The COVID-19 pandemic has worsened the situation of families struggling with dementia care in LACs, by delaying diagnosis and by increasing the burden on caregivers.^6,7^ Approximately 55% of the total population in LACs have informal jobs that demand leaving home to work and that often have no proper sheltering,^6^ which increases the risk of contagion for individuals with dementia. The reduction in medical appointments compromises care for dementia and other comorbidities, and quarantine can also increase the exposure of patients with dementia to mistreatment by family members.

LACs are experiencing a rapid demographic shift.^1^ The proportion of the population older than 60 years is rapidly increasing, and with it, the prevalence of dementia and the need for caregivers.^45,46^ Yet, caregivers are left to provide dementia care with no formal societal support and with a substantial financial strain. Their burdens are exacerbated by the stigma of dementia and of caregiving, delayed diagnosis, and currently by the COVID-19 pandemic.^6^ Without comprehensive health-care systems, social protection, and support for people with dementia and their caregivers,^8^ these economic and social burdens will grow exponentially. To alter this trajectory, proactive and coordinated strategies that integrate health and social care systems must be applied urgently. This is of crucial relevance because governmental systems for health and social protection in the region operate as separate systems. In most of the countries in the region, the two systems are minimally integrated,^5,47,48^ undermining adequate support for dementia caregivers.

## Short-term regional strategies

In response to the major challenges reviewed here, we propose seven short-term (3–5 years) initiatives both to alleviate burdens on caregivers and to identify their needs that are being met and those that are not. These initiatives would make use of the emerging multinational LACs initiatives^49–51^ to strengthen the local capacity to address caregiver needs.

### Action 1: set up systems to assess caregiver needs and resources

To provide adequate interventions and assessment of needs, systematic and validated instruments for regional assessment of caregiver burden are a crucial first step. Structured processes are required for the identification of needs and resources of caregivers in the diverse regions and cultures across LACs (comparing the current situation of dementia caregiving, summarised in the table, with the ideal scenario). Clinicians often ignore the caregiver and neglect to ask about their wellbeing, mood, or perceived burden. Therefore, a detailed guide for physicians about what to ask or how to test for and address caregiver burden in the clinic is important. The UNDP provides guidelines indicating how these assessments might be done in different situations, and how programmes and projects might be better designed to ensure their implementation, sustainability, and ultimately success.^52^ Despite similarities across the region, interventions need to be tailored and championed by regional leaders to suit the specific needs of the different cultures present across LACs. In addition, the assessment of caregiver needs requires a greater integration of different health-care sectors, including specialised dementia care, primary care, and social development divisions.

### Action 2: evaluate the effectiveness and implementation of evidence-based interventions for caregivers

Current initiatives within LACs are focused on providing economic support, diminishing caregiver burden (eg, by providing respite care, interventions to strengthen resilience, optimism and mindfulness, and cognitive behavioural therapy^10,53,54^), stabilising family dynamics (eg, through education programmes, monitoring, and community support^10,16,25,28^), or advocating for formal long-term care (eg, by promoting partial governmental support and regulations development^14,15,31^). A systematic review of methodologically robust studies on the effectiveness of caregiver interventions in LACs is still needed.^42^ In addition, the implementation of effective interventions has been a major challenge.^55^ The pragmatic clinical trial design might be a useful strategy to accelerate research on effectiveness and implementation, focusing on scalable interventions. This design can systematically and simultaneously examine outcomes and implementation of dementia care interventions.^56^

### Action 3: adapt to heterogeneous settings and cultures

Caregivers’ interventions in LACs need to be tailored to different settings and timelines (from diagnosis to end of life support).^34^ As recommended by WHO^57^ and by regional dementia plans,^2,46,58^ interventions should be tailored to primary health-care settings with input from specialists, and should include a social care plan. Although public health policies in LACs are driven by WHO’s Alma-Ata Declaration, which identifies primary health care as the main vehicle for achieving “health for all”,^58^ several LACs have undergone health-care privatisation. Unfortunately, these interventions to support caregivers have been poorly coordinated with social care services.^59^ As a consequence, caregivers navigate through fragmented care services, instead of having access to a case manager using evidence-based practice guaranteeing continuity of care.^60–62^ In a region characterised by social and health-care fragmentation and by a mixture of public and private health-care providers, case managers are highly needed.^59^ Additionally, specific interventions are needed for caregivers of patients with young-onset dementia and more complex dementia syndromes, such as frontotemporal dementia.^24,63^ Similarly, the needs of caregivers are often contingent on unique circumstances, such as whether they live in an urban or rural setting, or belong to an Indigenous community.^64^ The age of the caregiver is also an important consideration, especially if caregivers are themselves older spouses with greater susceptibility to health problems. Although research from LACs is still scarce, systematic reviews from different areas show how older age negatively affects caregivers’ access to information, training, management of medication, health, and adaptability.^65,66^ Thus, the combination of gender bias and age can further increase caregivers’ susceptibility to physical and mental health issues. The culturally embedded sense of duty towards older family members perceived by caregivers, which is particularly common in LACs,^10^ also needs to be considered when designing or adapting interventions.

### Action 4: reduce cultural gender stereotypes of care

As detailed in the studies presented in the table, dementia caregivers are disproportionately women (wives, daughters, and daughters-in-law) across the region.^8,15^ Care work is also combined with household tasks, role captivity (a feeling of absence of, or no freedom in the role of caregiver), loneliness, and financial strain. Governmental financial reimbursement and social care policies are potential mechanisms to reduce financial and emotional stress. Tailored support is crucial to combat gender stereotypes of care in LACs, and must consider the religious beliefs, low education and economic resources, and low level of access to information and to adult daycare of patients with dementia. Private and public societal engagement is also needed to change cultural beliefs about gender roles and stereotypes.

### Action 5: promote community and intergenerational support

Alzheimer’s disease associations and related organisations are available in almost all LACs. These non-profit organisations support people with dementia and their families. They have an essential role in disseminating relevant information locally, increasing prevention and promoting awareness of dementia, and fighting discrimination of both people with dementia and their caregivers. Other local community organisations in LACs, such as churches and clubs, can partner with dementia-oriented organisations to provide coping strategies for caregivers. Successful local support groups that achieve stress reduction, facilitate problem-solving, promote confidence, and maintain social interaction among caregivers^67^ should be further promoted and scaled up to national and regional levels. Mindfulness-based interventions are also potentially scalable (because they can be accessed through a smartphone) and can decrease caregiver burden, stress, anxiety, and depression.^68,69^ However, these interventions should be considered in combination with treatments for depression and other neuropsychiatric symptoms, as well as with assessments of vulnerable situations, an obstacle that is imperative to overcome. Common repositories of resources that can be useful for caregivers in different LACs are needed. For instance, in Colombia, the workshop series Cuidarte Cuidarme (Spanish for “take care of you, take care of me”), which was entirely virtual in 2020, continuously provides resources for caregivers.

### Action 6: ensure access to basic technological support

LACs still face multiple challenges in basic telecommunication infrastructure, especially in rural areas.^70^ Local governments and private corporations should support internet access as part of their social responsibility for caregivers and for reduction of the dementia burden. Digital resources can be a major aid for caregivers in rural areas or remote towns. Broadband internet can connect patients and their caregivers to hospitals, clinics, online peer support groups, and online social groups for dementia caregivers.

### Action 7: leverage technology to increase access to care

International initiatives offer promising support for caregivers,^71^ but they still need to be tailored to meet caregivers’ needs and to make them accessible for people with different technology literacy levels.^72,73^ Telephone counselling for dementia caregivers is a promising intervention that can be further exploited in LACs, especially in pandemic and postpandemic times.^6,74^ Telecommunication technologies (ie, tools and platforms for information sharing, promoting interventions, care coordination, and identification of people who have multiple vulnerabilities) can also assist during transitions of care, enabling continuity for the caregiver–patient dyads.^41^ Ongoing research initiatives across LACs, such as the Alzheimer’s Disease Neuroimaging Initiative, the Dominantly Inherited Alzheimer Network, the Worldwide FINGER network, and the Multi-Partner Consortium to Expand Dementia Research in Latin America, provide platforms for implementation science in the form of information and communications technology solutions for caregivers in the region.

## Long-term global scalability: brain health diplomacy and innovation tools

Despite the substantial and growing challenges faced by dementia caregivers, no coordinated regional responses exist currently.^2,6^ Most intervention research in LACs has been done in small samples and has not considered the influence of the regional landscape. Initiatives such as the FINGER network,^51^ based on multidomain lifestyle interventions to reduce risk of dementia, should be further paralleled by and focused on caregiver interventions in LACs. Considerable work on the implementation of effective and scalable interventions is needed. Advocates for caregiver support (caregivers, families, dementia-focused communities, and policy makers) do not have national platforms to coordinate efforts or develop effective initiatives. Moreover, LACs are very heterogeneous, and successful interventions in one place might not work in different circumstances (ie, low-resource *vs* high-resource, public *vs* private, and urban *vs* rural settings) without adaptation; inequalities and cultural differences must be considered in all regional plans. A multinational long-term strategy across LACs is essential for the development of lasting changes.^1,2,5^

Brain health diplomacy and convergence science^75,76^ can provide an innovative framework to design caregiver interventions in the context of inequalities, based on the integration of tools, knowledge, and strategies developed at the interface of multiple fields. Brain health diplomacy is an initiative transcending disciplinary boundaries that provides innovative scalable resources that improve brain health. Brain health diplomacy relies on different frameworks, including health diplomacy, science diplomacy, innovation diplomacy, and convergence science.^77^ Compared with classic isolated approaches, brain health diplomacy can better coordinate multisectoral actions by developing integrated strategies that directly tackle the challenges of caregivers.

Beyond the scope of traditional disciplines, brain health diplomacy can, in conjunction with LACs governments and non-governmental organisations, help to adapt these innovative solutions, with telemedicine, big data, and artificial intelligence, without ignoring the essential human factor, in a context of economic and infrastructure restrictions.^75,76^ Brain health diplomacy can bring translational support for dementia care management by use of scalable digital technology to reduce the costs and burden of dementia.^77^ Massive data monitoring and caregiver dyad health assessments can help to identify unmet needs and monitor response to interventions.^77^ However, maximising scalability requires coordinated actions at micro (individual), meso (community), and macro (national and transnational) levels via global policies to ensure improved outcomes. Brain health diplomacy should influence international diplomacy, not only at a local but mainly at a global level. Brain health problems cannot be solved solely by brain sciences, and brain health diplomacy provides an innovative approach to bring together solutions and coordinate disciplines and sectors.^75,76,78^ The advantages of this innovative approach became evident while assessing the effect of dementia in caregivers. Clinical interventions and training, financial strategies and investment relocation, policy regulations, public–private partnerships, and international support all need to be articulated at a transdisciplinary level to guarantee the success of long-term actions. Moreover, these coordinated actions should be integrated at both national and regional levels. Brain health diplomacy can also support the sharing of knowledge, strategies, and actions across countries with different capacities to face challenges in dementia (ie, countries with *vs* without national action plans or specific actions for caregivers).

Numerous studies in the USA and Europe have shown that collaborative care models for dementia successfully reduce caregiver burden.^79^ These multidisciplinary models provide both medical and supportive care, with special attention to caregiver needs.^80^ Even in high-income countries, however, translating these effective models into practice has proved challenging,^55^ and creative, scalable, and affordable approaches are needed,^81^ particularly in diverse and low-resourced LACs. A major cost-saving opportunity would be to train informal health-care workers to provide patients with access to appropriate care under the supervision of dementia specialists.^82,83^ Mobile phone and telephone-based care, either to supplement or replace clinic-based care, can reduce costs and burdens on families who might find it difficult to travel to a clinic appointment; the Care Ecosystem randomised clinical trial^84^ combined these approaches. For this telephone and web-based intervention, care team navigators were the primary point of contact for dementia families and delivered collaborative care from a centralised hub. The Care Ecosystem trial showed benefits for caregiver and patient wellbeing while reducing emergency-related health-care use.^84^ To succeed, brain health diplomacy needs to build strong links with ongoing and future governmental dementia plans to ensure that such coordinated initiatives are embedded in science-based policies. Ensuring that the Care Ecosystem, adapted through brain health diplomacy actions, can be generalised, globally connected, and locally adapted requires a formal commitment to brain health diplomacy from both local and global stakeholders. Thus, the development of global long-term strategies to scale up collaborative dementia care, although truly challenging, is essential to support the rapidly growing number of dementia caregivers in LACs, and to in turn reduce the vast inequalities across caregivers in the region.

## Conclusions

Major coordinated initiatives are needed to address the enormous burdens facing dementia caregivers in LACs. There is a need to test innovative, evidence-based short-term and long-term solutions to the major challenges, and such solutions must include well defined guidance for caregivers and involve all relevant stakeholders. Organisations such as the Global Brain Health Institute, which are pioneering approaches for both patients and caregivers with a particular emphasis in LACs, might favour further triangulation among global initiatives, emerging regional leaders, and available public policies. Efforts to unify LACs around dementia caregiving, as well as to advocate for international programmes that can be made widely available to all communities, will have an important effect on the care of patients with dementia and their caregivers.

## Figures and Tables

**Figure:**
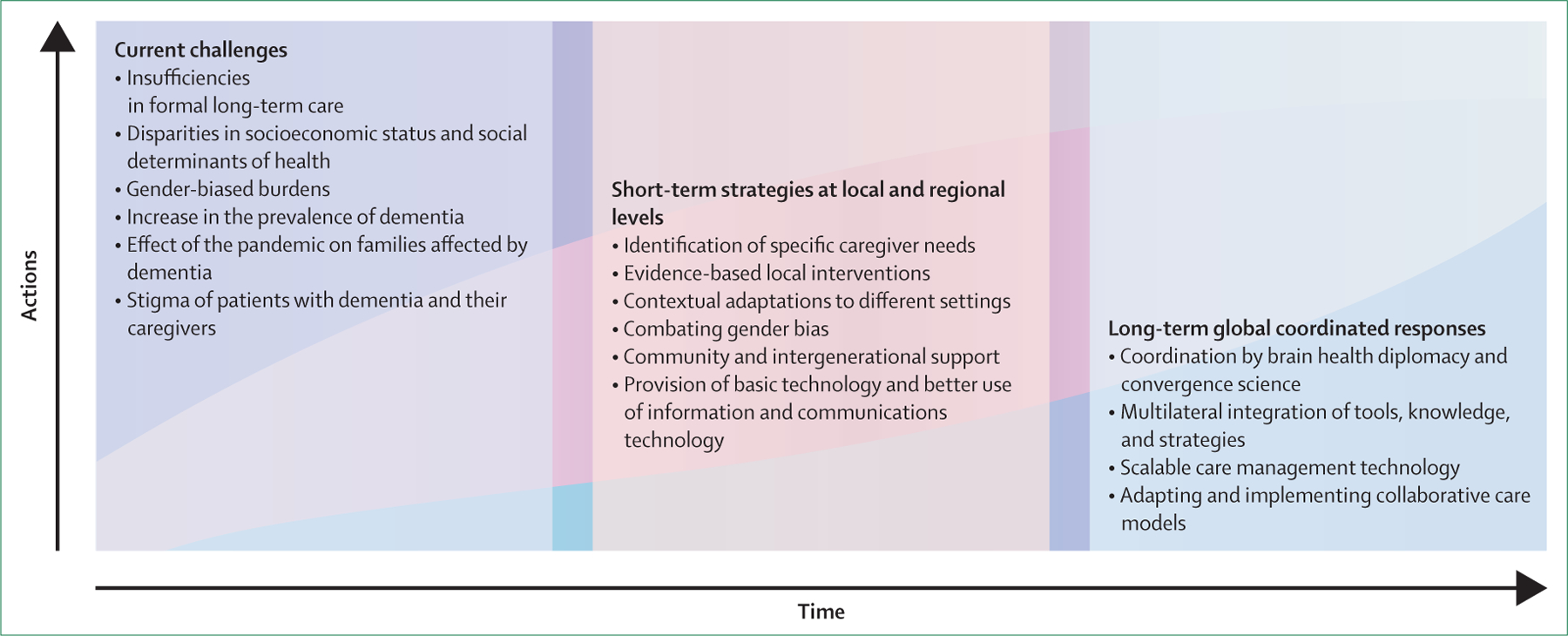
From challenges to global responses in dementia caregiving Timeline and workflow of current challenges, short-term strategies, and long-term responses for dementia caregiving.

**Table:** Studies of challenges to dementia caregiving in LACs

	Study design or focus	Location	Main results in LACs	Take-home message
Caruso et al (2017)^14^	LTC services	LACs	About 1% of the population over the age of 60 years lives in nursing homes and formal LTC is not a priority (in comparison with other requirements of public policies in the region); informal LTC is delivered disproportionately by women	Better LTC policies and normative framework are necessary
González et al (2014)^15^	Current initiatives for caregiving	LACs	Caregivers have poor access to medical care and social programmes; interventions target unpaid caregivers	There is insufficient capacity building (interventions that produce sustained change at individual to national levels) for caregiving; multicomponent interventions and an emphasis in gender needs and equality are necessary
Prince et al (2012)^8^	Multicountry study of caregiver profiles	LACs, India, and China	LACs have the highest caregiving burden; paid caregivers are common only in Cuba, Venezuela, and urban areas of Peru	Cultural barriers and resource availability limit remunerated support to caregivers and respite care, increasing the burden on unpaid caregivers
Prince (2004)^9^	Cross-sectional study	LACs, Asia, and Africa	31% of caregivers are older than 65 years, 65% are unemployed, and 64% are depressed; single caregivers are often in charge of caring for multiple generations; caregivers spend a mean of 6 h per day with the patient	Caregivers face adverse social, economic, and mental health conditions
Elnasseh et al (2016)^16^	Cross-sectional study	Argentina	Familial empathy determines resilience of caregivers and family communication increases the sense of coherence among caregivers	Personal strengths reduce caregiver stress and family interventions are required to support caregivers
Morlett Paredes et al (2017)^17^	Epidemiological study	Argentina	Worse cognitive function in individuals with dementia is associated with higher caregiver burden, depression, and anxiety	Patients’ mood and caregiver burden affect quality of care of caregivers; mental health and cognitive interventions are required
Allegri et al (2007)^18^	Cross-sectional study	Argentina	Caregiving burden increases with dementia severity and comorbidities (as reported by 88% of caregivers); 41% of caregivers left work or decreased their workload; indirect costs take 8 h per day of caregiver time	Dementia progression limits caregivers’ time for paid work; financial strain increases the burden
Rojas et al (2011)^19^	Cross-sectional study	Argentina	Across Alzheimer’s, frontotemporal and vascular dementia subtypes, most caregivers are spouses; higher costs of caregiving are associated with depressive symptoms and functional impairment in caregivers	Frontotemporal dementia causes higher caregiving costs than does Alzheimer’s disease and vascular dementia
Sutter et al (2016)^10^	Cross-sectional study	Argentina and Mexico	Personal strengths explained 32–50% of the variance in caregiver mental health in a sample of 127 primary family caregivers	Personal strengths are relevant to caregivers’ mental health; personal strengths and mental health approaches might partly compensate for the burden
Trapp et al (2015)^20^	Cross-sectional study	Argentina and Mexico	Personal strengths, including resilience, optimism, and a sense of coherence were associated with better mental and physical HRQOL	Interventions based on personal strengths and sense of coherence are recommended
Ferretti et al (2018)^21^	Cross-sectional study	Brazil	Caregivers had a mean of 9·43 (±5·68) years of education; substantial burden to private household expenditures due to dementia; dementia costs were influenced by the educational level of the caregiver	Low education increases dementia costs; education programmes can decrease financial burden
Gratão et al (2010)^22^	Cross-sectional observational study	Brazil	49 (54%) of 90 caregivers (of whom 80% were women) did not receive assistance (formal or informal); emotional burden was higher at the early and late stages of dementia	There is insufficient support for caregivers; the emotional burden on caregivers is higher in the early and late stages of dementia
Laks et al (2016)^23^	Cross-sectional study	Brazil	Caregiving is associated with psychiatric symptoms, high rates of presenteeism-related impairment, and work impairment	There is a need to develop support programmes, including for health care of caregivers and prevention of chronic non-communicable diseases in caregivers
Lima-Silva et al (2015)^24^	Cross-sectional study	Brazil	Caregivers of patients with behavioural variant frontotemporal dementia have more neuropsychiatric symptoms and distress than caregivers of patients with Alzheimer’s disease; dementia, anxiety, and depression positively correlate with burden in behavioural variant frontotemporal dementia caregivers	Caregiver distress is higher in families affected by behavioural variant frontotemporal dementia than by Alzheimer’s disease
Nogueira et al (2014)^11^	Cross-sectional study	Brazil	13% of male caregivers and 58% of female caregivers reported moderate to severe sexual dissatisfaction; impaired awareness and lower QOL of people with dementia is related to lower QOL of caregivers	QOL of caregiver not related to gender; lower QOL of people with dementia were related to the spouse’s QOL
	Topic	Location	Caregiving characteristics in LACs	Take-home message
Santos et al (2013)^25^	Cross-sectional study	Brazil	Disease awareness in caregivers of people with dementia and coping strategies were influenced by familism, religiosity, and duty	Religious beliefs relate to the importance of caregiving; cultural differences in norms and beliefs should be considered for caregivers’ interventions in the region
Sousa et al (2016)^12^	Cross-sectional study	Brazil and Spain	Caregivers’ sex, attendance of daycare centres, and neuropsychiatric symptoms had different effects in Brazilian and Spanish caregivers’ burden	Caregiver burden differed between Spain and Brazil; there are cross-cultural differences in caregiving
Hojman et al (2017)^26^	Cross-sectional survey	Chile	The highest cost of productivity loss is in female informal caregivers from low socioeconomic backgrounds; indirect costs (74%) are higher than in high-income countries (40%)	Socioeconomic status is inversely related to dementia care costs; socioeconomic status-related costs determine caregivers’ burden
Slachevsky et al (2013)^27^	Cross-sectional survey	Chile	Severe burdens were reported by 184 (63%) and psychiatric morbidity was found in 137 (47%) of 292 informal caregivers	Most caregivers are women and exhibit neuropsychiatric symptoms and functional impairment
Moreno et al (2015)^28^	Cross-sectional study	Colombia	102 informal caregivers presented poor mental health symptoms and reduced HRQOL	Culturally appropriate interventions should focus on preventing and treating depression and promoting life satisfaction of caregivers
Lloyd-Sherlock et al (2018)^13^	Qualitative family case study	Mexico and Peru	Family caregivers do not usually have days off; unmarried daughters and wives are typical caregivers; wives might require permission from a husband to provide care to another family member with dementia	Cultural norms and values are gender-biased and increase the caregiver’s burden
Mayston et al (2017)^29^	Household studies (LACs, Asia, Africa)	Mexico and Peru *vs* other countries	Health-care systems prioritise acute illness management; external support is viewed as temporary and paying family members is preferred	Governmental support is insufficient, private costs are unsustainable, and long-term care capacity is unmet
Custodio et al (2015)^30^	Retrospective cost study	Peru	People with low socioeconomic status cannot afford care in private settings; the total costs to families of frontotemporal dementia are higher than those of Alzheimer’s disease and vascular dementia due to caregiver demands	Reductions in family income increase caregivers’ burden; costs vary according to dementia subtype
Matus-López et al (2016)^31^	LTC policy	Uruguay	Domiciliary LTC is underfunded; public and daycare LTC are very scant; there is no financial aid for private care	LTC facilities and services are scarce and non-privileged; unpaid caregivers do not have specific training

HRQOL=health related quality of life. LACs=Latin American and Caribbean countries. LTC=long-term care. QOL=quality of life.

## Search strategy and selection criteria

Selective references for this work were identified by searching the PubMed database between Oct 1, 1997, and Dec 28, 2020. The search was complemented by other sources (Scopus and Google Scholar). The search terms combined relevant keywords (ie, “Latin America”, “Latin American countries”, “South America”, “Caribbean”, “Argentina”, “Bolivia”, “Brazil”, “Chile”, “Colombia”, “Costa Rica”, “Cuba”, “Dominican Republic”, “Ecuador”, “El Salvador”, “Guatemala”, “Honduras”, “Mexico”, “Nicaragua”, “Panama”, “Paraguay”, “Peru”, “Uruguay”, or “Venezuela” each in combination (and) with “caregiving”, “carers”, “care” (and) with “aging”, “dementia” (or) “neurodegeneration”, “neurodegenerative diseases”, “older adults”, “brain health”, “Alzheimer’s disease”, “frontotemporal dementia”, “Lewy body dementia”, “Parkinson’s disease”, and “vascular dementia”). Key concepts for the search included dementia prevalence, health care, regional inequalities, caregiver burden, long-term care, social determinants of health, socioeconomic status, gender bias, impact of COVID-19 on dementia, caregiver needs, evidence-based interventions, and available communications technology. References assessing brain health diplomacy and caregiving interventions, platforms, or initiatives were also scrutinised by combining the different search terms. Only papers published in English were included in the final version of the present work, although references in Spanish and Portuguese were also reviewed. Additional pertinent references related to the topics discussed were also selected by the authors and included in the manuscript.

## Panel: A first-person view of caregiving in Latin America

Storytelling can be a powerful tool for behaviour changes regarding dementia caregivers. Here we provide a first-person story from a caregiver and coauthor of this work (FAP) who specialises in narratives as a tool to develop more comprehensible knowledge for caregivers in Latin America.

I was a teenager in Brazil when my grandmother and best friend was diagnosed with Alzheimer’s disease. It was a dark time. My father was unemployed, and my mother was depressed. I went through the most painful process during the disease, struggling to accept an incurable diagnosis and pushing my capabilities to their limits. I often felt overwhelmed, and that I was not fulfilling my duty as a grandson. I felt like a passive observer of her decline. We felt alone in these challenges and had great difficulty communicating with health professionals in our public health system. We suffered from our community’s lack of awareness and of acceptance of dementia. We are still living the consequences of the cultural association of ageing and disability. A large proportion of people in Latin American consider ageing as a purely incapacitating process. There is a nickname for old people in Brazil: caduco (expired), which reinforces stigma and associates old age with memory loss, language issues, and social isolation as inevitable ageing-related processes.

Caregivers should be empowered and supported to have an active role in guiding dementia care. Policies and models of care need multidisciplinary therapies to decentralise medical actions and to mitigate feelings of helplessness. Physiotherapy, speech, occupational, and other non-pharmacological therapies are needed to reduce caregiver burdens. Family caregivers and people living with dementia should be included in elaborating new strategies, and should be considered co-decision makers. The inclusion of families in public policy registries is essential for tailored assistance and monitoring of assessments and interventions.

During the COVID-19 pandemic, I relived many emotions from when I cared for my grandmother. The pandemic has only worsened the fears and challenges already existing in the lives of many families facing dementia. Social isolation, widely discussed in public policy and social media debates, was my daily life as a caregiver. The scarcity of care resources, public policies, and awareness amplified my grandmother’s and my own isolation. Many family members—especially daughters and wives—give up their jobs, compromise their relationships, and have their social life and self-care negatively affected. Perhaps for the first time, the world is empathically connected with the daily challenges of caring for someone with dementia in Latin America: the fear of tomorrow, the difficulty of communicating with the outside world, and the physical and emotional isolation.

It is time to accept and respect these families, leading them to a healthier way of caring. Empowering families by highlighting their unique role in maintaining dignity will bring more inclusive and achievable postdiagnostic strategies for those facing dementia.
